# No Evidence of Gut Microbiota Alteration in Psoriasis Patients Switching to Brodalumab after Loss of TNFα Inhibition Effect

**DOI:** 10.3390/ijms25147745

**Published:** 2024-07-15

**Authors:** Admir Vižlin, Ylva Andersch Björkman, Yadhu Kumar, Maria Göthe, Martin Gillstedt, Amra Osmančević

**Affiliations:** 1 Department of Dermatology and Venereology, Institute of Clinical Sciences, Sahlgrenska Academy, University of Gothenburg, 413 45 Gothenburg, Sweden; admir.vizlin@gmail.com (A.V.);; 2 Department of Dermatology and Venereology, Sahlgrenska University Hospital, Region Västra Götaland, 413 45 Gothenburg, Sweden; 3 Eurofins Genomics, 78467 Konstanz, Germany

**Keywords:** psoriasis, depression, gut microbiota, brodalumab, TNFα

## Abstract

Biological agents used to treat severe psoriasis may alter the gut microbiota, though current knowledge is limited. This study examines whether switching from TNFα inhibitors, from which patients had reduced or lost effect, to brodalumab, an IL-17 inhibitor, affects the gut microbiota in patients with psoriasis and how these changes correlate with the clinical variables of psoriasis severity and depressive symptoms. Fecal samples from patients were collected before the treatment switch and 12 weeks after the switch and were analyzed for the microbiota composition using next-generation sequencing targeting the V3–V5 region of the 16S rRNA gene, followed by bioinformatics analysis. No significant changes in overall gut microbiota composition were observed after the treatment switch, although individual variations in the Firmicutes/Bacteroidetes ratio were noted, and no significant correlations with clinical variables were found. These findings suggest that short-term changes in gut microbiota in patients with psoriasis are limited and that dysbiosis may be influenced by the interplay of various microbial populations rather than specific taxa. This study provides a foundation for further research into the effects of biological treatments on the gut microbiota in patients with psoriasis.

## 1. Introduction

Psoriasis is an immune-mediated disease characterized by scaly patches, affecting 2–3 percent of the global population [1]. It is associated with various comorbidities, notably depression [2], a psychiatric condition marked by low mood and loss of interest, with a prevalence of 3.2% in patients without comorbidities and up to 23% in patients with comorbidities [3]. Both psoriasis and depression have a bidirectional relationship, with systemic inflammation playing a significant role [4]. The gut microbiota has an important role in regulating the immune system and maintaining homeostasis, therefore aiding in preventing systemic inflammation [5]. 

Psoriasis and depression are diagnosed based on clinical presentation. The two most commonly used assessment tools are the Psoriasis Area and Severity Index (PASI) and the Dermatology Life Quality Index (DLQI) [6,7]. PASI grades the severity of erythema, induration, and scaling, while DLQI assesses the impact a dermatological disorder has on the quality of life and psychological well-being. Mild psoriasis is defined as PASI 3 and DLQI ≤ 5, and moderate psoriasis falls within PASI 3–9 and DLQI 6–9, while severe psoriasis is defined as PASI ≥ 10 and DLQI ≥ 10, according to Swedish guidelines [8]. Depression is usually assessed using the Montgomery-Åsberg Depression Scale (MADRS) and the Hospital Anxiety and Depression Scale (HADS) [9,10]. 

Research indicates that patients with psoriasis or depression exhibit gut microbiota dysbiosis compared to healthy controls, suggesting a partial loss of the microbiota’s anti-inflammatory effects [11]. Interestingly, systemic inflammation from gut microbiota dysbiosis, which could be a result of both psoriasis and depression, stems from various factors, one of which is a decrease in short-chain fatty acids (SCFAs) [11]. This will eventually cause the gut–blood barrier to become leaky, resulting in impaired antigen-presenting abilities and the translocation of bacteria and their endotoxins into the bloodstream, including lipopolysaccharides (LPSs) [11]. They can be detected by Toll-like receptors, more specifically the TLR4/MD-2 complex, which is expressed on the surface of macrophages, monocytes, dendritic, and epithelial cells and can sense picomolar concentrations of LPS, resulting in the release of various cytokines, such as interleukins (IL), IL-1, IL-6, and Tumor Necrosis Factor α (TNFα) [11,12]. This will induce the differentiation of T-helper cells (Th), mainly Th17, which will induce the production of IL-17A. Lastly, IL-17A will trigger the abnormal proliferation of keratinocytes in psoriasis while simultaneously activating microglia and astrocytes in the central nervous system, resulting in neuroinflammation, synaptic dysfunction, and reduced serotonin levels due to the breakdown of tryptophan, contributing to depression [11]. However, these pathways have only been seen in experiments with rodents and are not well confirmed in human studies yet [11]. 

The human gut microbiota consists predominantly of six phyla, Bacillota (Firmicutes), Bacteroidota (Bacteroidetes), Actinomycetota (Actinobacteria), Pseudomonadota (Proteobacteria), Fusobacteriota (Fusobacteria), and Verrucomicrobiota (Verrucomicrobia), which can be further divided into various genera and species [13,14]. Interestingly, various taxa have been shown to be altered in patients with psoriasis compared to healthy controls, such as the phyla Bacillota (Firmicutes), Bacteroidota (Bacteroidetes), Actinomycetota (Actinobacteria), and the genera *Blautia* and *Faecalibacterium*. Notably, an increased Bacillota/Bacteroidota (Firmicutes/Bacteroidetes) ratio is linked to dysbiosis of the gut microbiota, which patients with psoriasis express [15]. Other studies have shown various results, such as *Blautia*, *Bifidobacterium*, and *Ruminococcus* increasing and *Bacteroides*, *Alistipes*, *Paraprevotella*, and *Faecalibacterium* decreasing in patients with psoriasis compared to healthy controls [16]. On the contrary, there have also been studies displaying an increase in Bacteroidota (Bacteroidetes) and a decrease in Bacillota (Firmicutes) compared to healthy controls [17]. Additionally, an altered gut microbiota has been observed in patients with depression, displaying various taxa that differ from healthy controls [18,19]. The gut microbiota is dynamic and can be affected by various factors, such as sex, age, ethnicity, Body Mass Index (BMI), and enterotypes, which are defined as bacterial clusters that can be observed in three different ways (hence, three enterotypes). These enterotypes are stable through adulthood and can be restored if altered [13].

Biological agents, commonly used for severe psoriasis, have the potential to alter the gut microbiota. A systematic review of multiple biologics in different inflammatory conditions shows that the β-diversity did not change significantly after the use of either TNF-α or IL-17 inhibitors in patients with psoriatic arthritis [20]. Furthermore, TNF-α and IL-17 inhibitors did not show significant differences pre- and post-treatment in Bray–Curtis distances, indicating no change in the gut microbiota before and after these treatments [20]. In addition, other studies in the review have shown that patients treated with TNF-α inhibition displayed similar gut microbiota as healthy controls, showing that TNF-α inhibitors have the potential to restore the gut microbiota in a state of eubiosis or at least make it similar to the gut microbiota of healthy individuals [20]. Interestingly, patients with psoriasis on IL-17 and IL-12/23 inhibitors showed no changes in the short term. However, the use of secukinumab (an IL-17 inhibitor) showed distinct changes in the gut microbiota after 6 months of treatment [20]. Nonetheless, the role of the gut microbiota in preventing systemic inflammation and its effects on diseases like psoriasis and depression are not fully understood, and the impact of biological agents on the gut microbiota and their connection to clinical outcomes in psoriatic patients remains largely unexplored.

This study aimed to investigate how the composition of the gut microbiota presents and if there are any alterations before and after 12 weeks of switching treatment from TNFα inhibitors to brodalumab, an IL-17A inhibitor that is much broader than agents of the same kind, inhibiting numerous cytokines, including IL-17A, IL-17F, IL-17A/F heterodimer, IL-17C, and IL-17E [21]. We also aimed to explore correlations between changes in specific taxa and clinical variables related to psoriasis severity and depressive symptoms.

## 2. Results

### 2.1. Demographics

A total of 22 patients were initially screened, and 20 of them met the inclusion criteria for the study. Of those, 14 were men, and 6 were women, of whom 3 patients did not complete the study due to either joint pain (*n* = 1), traveling limitations (*n* = 1), or unspecific stomach problems connected to TNF-α inhibition (*n* = 1). In total, 17 patients completed the study. Patients were selected from the SAHL1011 study (Table 1) [22]. All patients, except one, responded to the treatment with brodalumab and improved in both their psoriasis severity, with PASI (*p* < 0.0001) and DLQI (*p* = 0.004) significantly improved, and depressive symptoms, with MADRS-S (*p* = 0.0007) significantly improved [22].

### 2.2. Raw Data

Raw sequencing data targeting the 16S rRNA gene were acquired from Eurofins Genomics (Konstanz, Germany). The dataset comprised a total of 149,760 to 160,000 reads per sample, with an average proportion of high-quality reads ranging from 85.8% to 89.9%. The sequence reads were further assembled to generate amplicon length sequences. The final amplicon reads exhibited an average length of 530 base pairs, with a minimum of 60,000 amplicon readouts per sample. Notably, the sequence quality remained consistent and of high quality across all samples.

### 2.3. Composition of the Gut Microbiota

**Figure 1:**
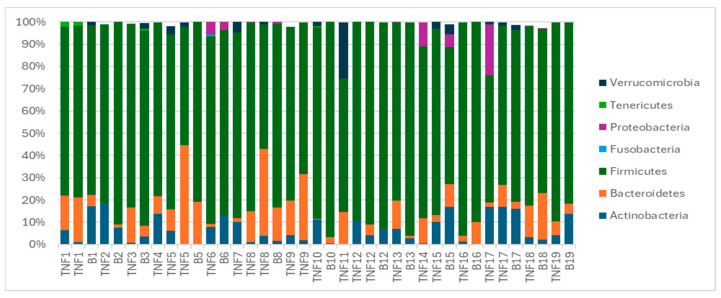
The bar chart presents the composition of the gut microbiota on the phylum level before (TNF) and after (B) the switch to brodalumab. Each number represents one patient (patients were numbered according to the recruitment order). Some patients took two samples before the switch, indicating three total samples. Some patients did not submit samples after the switch, hence the absence of five samples marked with (B), which were TNF4, TNF7, TNF9, TNF11 and TNF14. Each bar is a sample. The *y*-axis represents the relative abundance of phyla. The figure is descriptive, showcasing the gut microbiota profile of every sample and patient.

The gut microbiota mainly consisted of the three phyla: Bacillota (Firmicutes), Bacteroidota (Bacteroidetes), and Actinomycetota (Actinobacteria). Bacillota (Firmicutes) was the most common phylum across all groups. Figure 1 illustrates the microbiota profile before and after switching to brodalumab, depicting the gut microbiota composition in psoriasis patients pre- and post-treatment switch.

### 2.4. Differences of Groups before and after Treatment Switch

#### 2.4.1. Principal Coordinate Analysis

**Figure 2:**
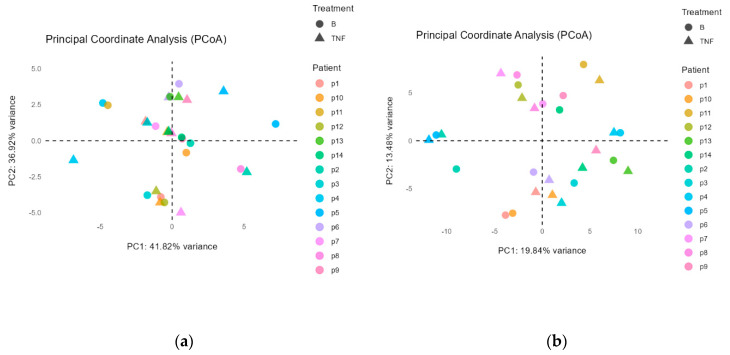
Principal Coordinate Analysis for (**a**) phylum and (**b**) genus. Triangles (TNF) represent the gut microbiota before the switch, and circles (B) represent the gut microbiota after the switch. Patient numbers were reassigned to only include patients who had submitted samples both before and after the switch. Only taxa with a relative abundance of at least 0.5% were included, with the total percentage of the variance showcasing the total amount of taxa exceeding that threshold. The closer the samples before and after the switch are, the more similar the composition of the gut microbiota is. Both plots show that the distance between samples before and after the switch is low, indicating that the gut microbiota did not change significantly after the switch on neither the phylum (*p* = 0.44) nor the genus (*p* = 0.67) level.

The Principal Coordinate Analysis revealed no significant changes in gut microbiota composition after switching to brodalumab at both the phylum (*p* = 0.44) (Figure 2a) and the genus levels (*p* = 0.67) (Figure 2b). However, significant differences were observed between patients at both the phylum (*p* = 0.002) (Figure 2a) and the genus levels (*p* = 0.001) (Figure 2b). This indicates that individual microbiota profiles remain unique and significantly different, with the treatment switch not causing significant compositional changes after 12 weeks.

#### 2.4.2. Differential Abundance Analysis

**Figure 3:**
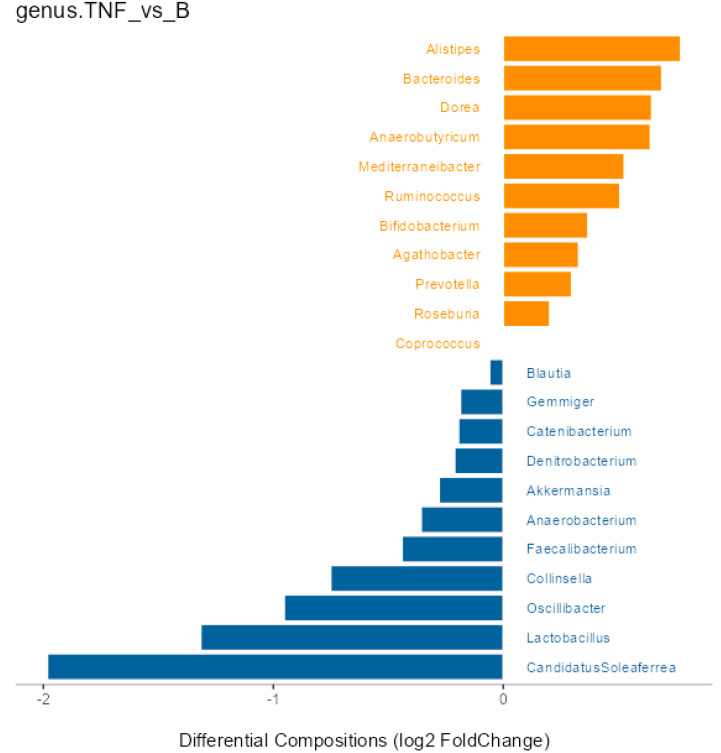
Differential Abundance Analysis at the genus level, only including genera with a minimal relative abundance of 0.5%. The plot represents taxa more abundant before the change (orange) and after the change (blue). This implies that the orange taxa decreased after the switch, while the blue taxa increased after the switch. None of these changes were significant (*p* > 0.05).

**Figure A1:**
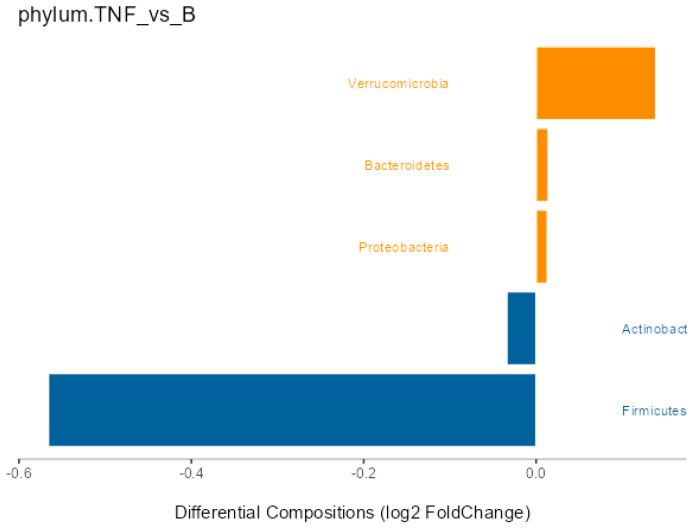
Differential Abundance Analysis at the phylum level, only including phyla with a minimal relative abundance of 0.5%. The plot represents taxa more abundant before the change (orange) and after the change (blue). This implies that the orange taxa decreased after the switch, while the blue taxa increased after the switch. None of these changes were significant (*p* = 0.96).

**Figure A2:**
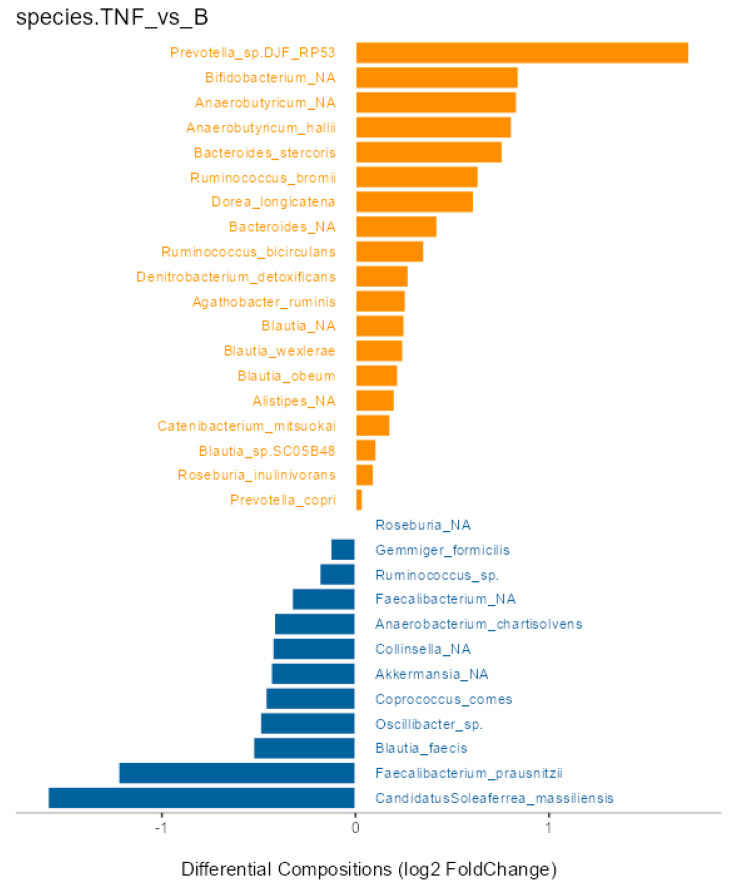
Differential Abundance Analysis at the species level, only including species with a minimal relative abundance of 0.5%. The plot represents taxa more abundant before the change (orange) and after the change (blue). This implies that the orange taxa decreased after the switch, while the blue taxa increased after the switch. None of these changes were significant (*p* > 0.05).

The Differential Abundance Analysis identified Firmicutes as the most altered phylum post-treatment-switch, with an observed increase that was not statistically significant (*p* = 0.21). Changes in other phyla were similarly insignificant (*p* = 0.96) (Figure A1). At the genus level, various taxa showed changes, none of which reached statistical significance (*p* > 0.05) (Figure 3). Likewise, the changes at the species level were insignificant (*p* > 0.05) (Figure A2). These findings suggest that while specific taxa may not significantly change, their interplay could still be relevant in psoriasis patients’ gut microbiota.

### 2.5. Beta Diversity Analysis

**Figure 4:**
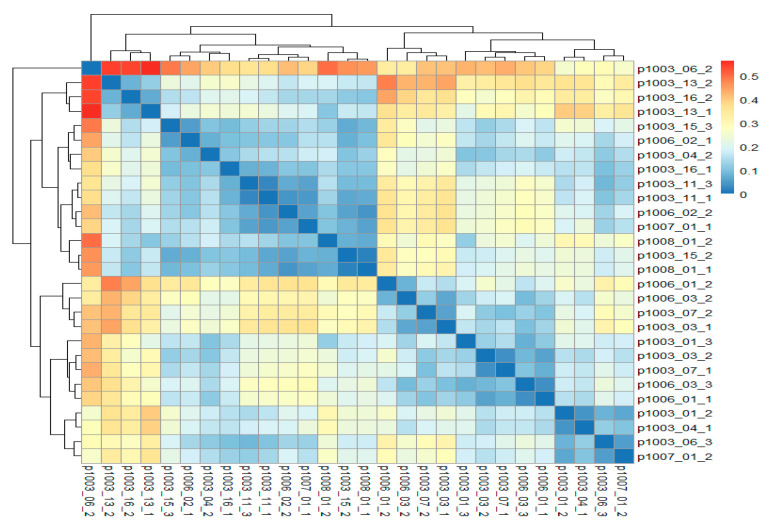
Heat map of the beta-diversity of phyla. Blue represents no difference between two samples, while red represents differences between two samples. Most samples do not differ greatly from each other, indicating and confirming that there is no evidence of alterations in the gut microbiota after switching treatment to brodalumab.

A heat map of beta-diversity demonstrated no significant differences at the phylum level in samples before and after switching to brodalumab (Figure 4), reinforcing the notion that the observed changes are not statistically significant. Although some samples differed significantly, the majority did not. Thus, there is no evidence that the gut microbiota would change after switching treatment to brodalumab.

### 2.6. Alpha Diversity Analysis

**Figure 5:**
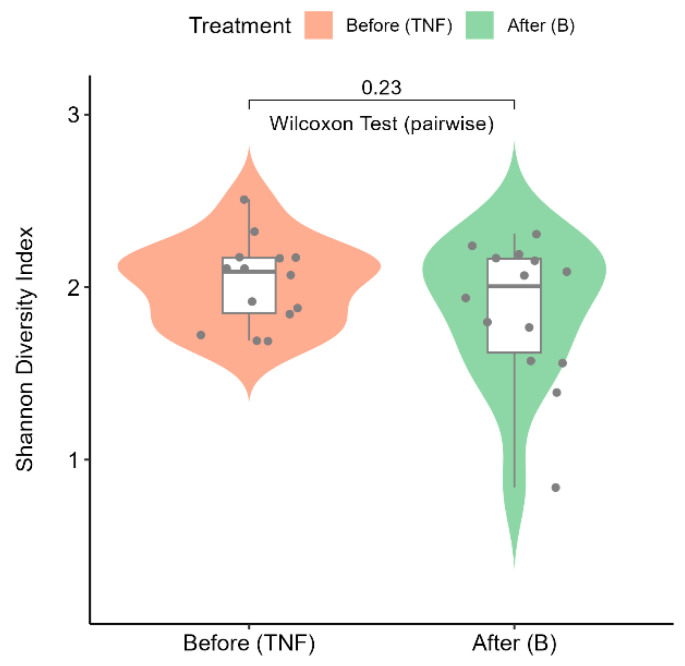
Violin plot showing the distributions of the Shannon–Wiener diversity indices across treatment groups. The pink violin represents diversity indices from patients before treatment (TNF), while the green violin represents diversity indices after treatment (B).

The Shannon–Wiener diversity index (alpha diversity) is calculated to quantify the diversity of microbial communities in patient groups before and after treatment. The violin plot (Figure 5) shows the distributions of the Shannon–Wiener diversity indices across treatment groups. The alpha diversity values are consistent between the treatment groups, as evidenced by the non-parametric pairwise statistical test (Wilcoxon signed rank test) performed on the two groups. The *p*-value of 0.23 indicates that no significant differences were observed due to treatment on microbial compositions before and after treatment.

### 2.7. Bacillota/Bacteroidota (Firmicutes/Bacteroidetes) Ratio

**Figure 6:**
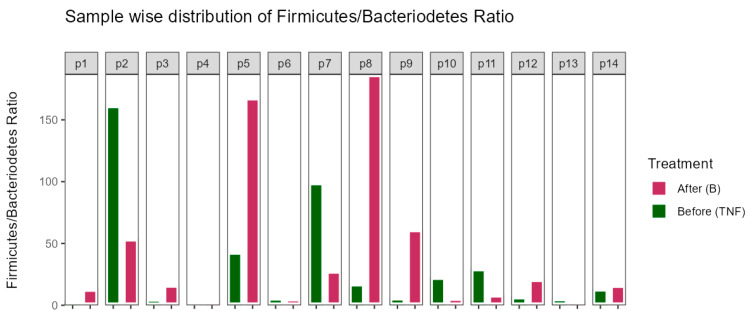
Bacillota/Bacteroidota (Firmicutes/Bacteroidetes) ratio before (green—TNF) and after (purple—B) switching treatment. Patients who showed the largest variations were p2, p5, p7, and p8. All the affected patients were responders, apart from p5, who was a non-responder to the switch.

Analysis of the Bacillota/Bacteroidota (Firmicutes/Bacteroidetes) ratio indicated no observable changes for most patients. However, some patients showed notable increases or decreases in this ratio. Such variations could indicate potential dysbiosis, suggesting that, despite the lack of significant changes in other analyses, some patients might experience shifts in their gut microbiota that could contribute to a dysbiotic state. This observation, while not definitive, highlights the potential for individual variability in response to treatment (Figure 6).

### 2.8. Correlation to Clinical Variables

The correlation of phyla and the Firmicutes/Bacteroidetes ratio to the clinical variables PASI, DLQI, MADRS-S, and HADS was conducted on the baseline values and showed no significant correlation after correcting for multiple testing (*p* = 1.00). This indicates that specific phyla do not change based on the values of clinical variables and are therefore not affected by the change in value in these variables (Table 2).

## 3. Discussion

Our study found that Bacillota (Firmicutes) and Bacteroidota (Bacteroidetes) were the predominant phyla across all groups. No significant changes in gut microbiota composition were observed after switching to brodalumab, indicating microbiota stability despite the treatment switch and, therefore, no evidence of alterations in the gut microbiota. However, some patients exhibited significant changes in their Bacillota/Bacteroidota (Firmicutes/Bacteroidetes) ratio, suggesting individual variability in response to treatment. No significant correlations were found between specific phyla and clinical variables.

We found that Bacillota (Firmicutes) was the dominant phyla regardless of the treatment switch, followed by Bacteroidota (Bacteroidetes). These findings align with studies by Wen et al., Shapiro et al., and Doaa et al., who examined untreated psoriasis patients [15,17,23]. Conversely, studies by Dei-Cas et al. (who looked into untreated psoriasis patients) and Valentini et al. (who looked into both untreated and biologically treated psoriasis patients) reported Bacteroidota (Bacteroidetes) as the dominant phylum, with Bacillota (Firmicutes) being the second most common [24,25]. These discrepancies could be due to differences in patient characteristics, unconsidered enterotypes, and varying sequencing approaches targeting different parts of the 16S rRNA gene. Nonetheless, Bacillota (Firmicutes) and Bacteroidota (Bacteroidetes) remain the two dominant phyla across all groups.

We observed no correlations between clinical variables for psoriatic and depressive symptoms and specific phyla. This contradicts the findings of Doaa et al., who found correlations between PASI and the Bacillota/Bacteroidota (Firmicutes/Bacteroidetes) ratio and Actinomycetota (Actinobacteria) [23]. The small sample size in our study likely contributed to these insignificant correlations. This suggests that lower taxonomical hierarchical levels might also lack correlation with clinical variables. Nonetheless, it gives some insight into the possible association between clinical variables and different phyla.

Patients responded well to treatment, with PASI and DLQI scores significantly decreasing after switching to brodalumab. However, whether the baseline gut microbiota can predict response to treatment is unknown and requires further studies to elucidate the possibility. In addition, deciding if changes are greater in responders versus non-responders, or vice versa, is difficult since our study only had one non-responder, making it impossible to draw relevant conclusions. Thus, further studies are needed to explore these possibilities.

Our study observed no significant changes in the gut microbiota 12 weeks after switching to brodalumab. Similarly, Zhao et al. reported no significant changes in the gut microbiota of psoriatic patients before and after TNF-α inhibition [26]. Interestingly, significant differences between psoriatic patients and healthy controls suggest that psoriatic patients may have dysbiosis, while healthy controls have eubiosis. In addition, the findings of Todberg et al. in their letter to the editor also state that the use of TNF-α inhibition did not induce changes in the gut microbiota in psoriatic patients compared to patients before treatment initiation [27]. Shapiro et al. and Hidalgo-Cantabrana et al. observed dysbiosis in psoriatic patients, and interestingly, Jiang et al. similarly reported dysbiosis in patients with active depression [15,16,18]. These results indicate that changes in the gut microbiota might not occur in the short term after a treatment switch due to its dysbiotic nature. Therefore, longer follow-ups are necessary to assess possible long-term changes.

Despite observing various phyla, genera, and species differences after the switch, none of these changes were significant in our study. This indicates that the switch did not alter the gut microbiota significantly. Considering the findings of the systematic review, IL-17 inhibitors seem to not induce significant changes in the gut microbiota in the short term but seem to do so after 6 months [20]. This aligns with our study regarding short-term changes. However, longer follow-ups are needed to assess if brodalumab can change the gut microbiota in the long term, as is the case with secukinumab. In addition, since previous studies in the review showed no significant gut microbiome changes before and after TNF-α or IL-17 inhibitors, it aligns with our findings that there were no significant changes in the gut microbiota after switching to brodalumab. However, further studies with larger sample sizes and longer follow-ups are needed to assess the true effects of brodalumab [20].

Our findings suggest that the Bacillota/Bacteroidota (Firmicutes/Bacteroidetes) ratio was significantly altered for some patients, while others experienced no change. Shapiro et al., Hidalgo-Cantabrana et al., and Dei-Cas et al. observed increased ratios in untreated psoriatic patients, possibly indicating dysbiosis and the depletion of SCFAs, potentially weakening the gut–blood barrier and contributing to low-grade inflammation [15,16,24]. Therefore, the patients who observed a decreased ratio (2 patients) after the switch in our study might suggest a positive effect on the gut microbiota, while the patients who had an increased ratio (2 patients) might suggest dysbiosis and worsening of symptoms due to the switch. Interestingly, one of the patients (p5) who experienced an increase in the ratio after the switch was a non-responder and had symptom exacerbations, suggesting that an increased ratio might be associated with treatment failure and worsened dysbiosis. However, this finding was observed in only one patient, necessitating further studies to validate this association. Nonetheless, brodalumab might have the potential to alter the Bacillota/Bacteroidota (Firmicutes/Bacteroidetes) ratio, but further studies are needed to assess the true effect. It is, however, important to emphasize that the ratio itself does not dictate if dysbiosis is prevalent or if a patient is at a higher risk of not responding to treatment, since the ratio itself is highly variable due to different factors. Rather, it is the interplay between all microbial populations that is crucial [28,29]. Nonetheless, the ratio does give some insight into the possible effects of the treatment switch.

Comparisons with previous studies reveal mixed results. Shapiro et al. reported increased abundances of *Blautia*, *Faecalibacterium*, *Ruminococcus*, and *Dorea* in untreated psoriatic patients, while our study found decreases in *Ruminococcus* and *Dorea* after the switch to brodalumab [15]. Hidalgo-Cantabrana et al. found increases in *Bifidobacterium* and *Ruminococcus* in untreated psoriatic patients [16], contrary to our findings. At the species level, Jiang et al. found that depressive patients had increased abundances of *Alistipes* and *Roseburia*, while *Bacteroides*, *Prevotella*, and *Ruminococcus* decreased [18]. Our study found decreases in all the previously mentioned genera, suggesting that brodalumab might influence depressive symptoms. Furthermore, Schade et al. found that untreated psoriatic patients expressed increased *Prevotella* and decreased *Ruminococcus*, *Blautia*, and *Akkermansia* [30]. Our study was in line only regarding *Ruminococcus*, while we observed the opposite for the other taxa. Interestingly, Scher et al. found that *Ruminococcus* decreased in untreated patients with psoriatic arthritis [31], raising the question of whether the reduction in the same genus in our study could contribute to a higher risk of developing psoriatic arthritis. Nonetheless, discrepancies between our results and those of other studies may stem from the fact that previous studies compared the gut microbiota of psoriatic or depressive patients to healthy controls, while our study did not include a healthy control group. This suggests that brodalumab or switching biological agents in general could either aid in restoring eubiosis or worsen dysbiosis. Since our observations were not significant, further studies are needed to assess which taxa differ after switching to brodalumab and if that alteration contributes to the eubiosis or dysbiosis of the gut microbiota.

Being a pilot study, it comes with several limitations. The absence of a control group made it impossible to differentiate how the gut microbiota differs in healthy controls, which is something to be included in further studies, especially when previous studies, such as Zhao et al., showed that the gut microbiota did not differ between psoriatic patients before and after treatment initiation but differed against healthy controls [26]. Additionally, incomplete sample submissions from some patients reduced the sample size and potentially affected the results. While 16S rRNA sequencing is precise, it covers a limited number of detectable taxa. Future studies should consider metagenome sequencing for comprehensive analysis. Despite limitations, the study methodology addressed the aim and aligned with previous research designs.

Given the limitations of the study, there are areas for improvement and potential other aspects to consider. Conducting metagenome sequencing would contribute to a better taxonomical picture, with the potential to explore how changes in the gut microbiota after treatment switch could affect the production of SCFAs. Furthermore, previous studies show that enterotypes could play a crucial role in how prone the gut microbiota is to become dysbiotic in psoriatic patients, especially regarding enterotype II [32]. Notably, enterotype II has also been shown to be dysbiotic compared to other enterotypes in depression [33]. Thus, it would be interesting to examine the correlations of specific taxa and clinical variables with different enterotypes and how they could be involved in how patients respond, or will respond, to treatment with biological agents. Extending follow-up periods and increasing sampling frequency, along with stratifying patients by psoriasis severity and enterotypes, could offer a more detailed understanding of the dynamics of the gut microbiota upon switching or initiating biological treatments.

## 4. Materials and Methods

### 4.1. Study Design

This pilot project was part of the broader phase 4 open-label randomized SAHL1011 study, involving both parallel groups and crossover setups. In total, 20 patients were selected based on specific inclusion and exclusion criteria between March 2019 and September 2021. Inclusion criteria required a minimum PASI score of 7 or PASI 3–7 combined with a DLQI > 5 and Body Surface Area (BSA) > 3. In addition, patients had to have received TNF-α inhibitors for 4–6 months prior to the start of the study and had to be at least 18 years old. Exclusion criteria included systemic diseases, including renal failure, heart failure, hypertension, liver disease, anemia, and diabetes; a history of inflammatory bowel disease (IBD); previous treatment with IL-17 inhibition; or suicidal behavior. Hypersensitivity to the administered drug also resulted in exclusion. 

Patients, who all responded poorly to TNF-α inhibitors, were divided into two groups: Group TNF/B (randomized with 8 patients), which continued with TNFα-inhibitors, and Group B, which switched to brodalumab 210 mg at baseline (randomized with 12 patients). Group TNF/B switched to brodalumab after 12 weeks. These groups were pooled into 2 categories of samples: before and 12 weeks after the treatment switch. All patients were instructed to collect fecal samples for further sequencing. Samples were collected on 3 occasions, depending on the assigned group: TNF/B took 2 samples before the switch (baseline and week 12) and 1 sample after the switch (week 24), while B took 1 sample before the switch (baseline) and 1 sample after the switch (week 12). All samples were stored at −80 degrees Celsius before further use upon collection [22]. In the TNF/B group, samples taken at week 12 (immediately before the switch) were used for paired statistical analysis (Principal Coordinate Analysis, Beta Diversity Analysis, Alpha Diversity Analysis, and Differential Abundance Analysis).

### 4.2. Sequencing and Evaluation of Clinical Outcomes

Sequencing and bioinformatics were conducted by Eurofins Genomics (Konstanz, Germany). The hypervariable V3–V5 region of the 16S rRNA gene was targeted for sequencing. DNA extraction utilized commercially available kits with optimized protocols, as detailed by Eurofins Genomics. Samples were crushed mechanically using a bead beating process and chemically using the lysis buffer that was supplied with the corresponding DNA extraction kit.

Afterwards, a two-step PCR-protocol was conducted. During the first step, the hypervariable V3-V5 region of the 16S rRNA gene was targeted using region-specific primers (forward: CCTACGGGNGGCWGCAG and reverse: CCGYCAATTYMTTTRAGTTT) [34,35], which included a universal adapter sequence (Illumina TruSeq adapter) for subsequent indexing. Following indexing, samples were pooled to give approximately 60,000 read pairs per sample and then loaded on the MiSeq flow cell (Illumina, San Diego, CA, USA) and sequenced on an Illumina MiSeq sequencer (Illumina, San Diego, CA, USA) with v3 chemistry in 2 × 300 bp mode. Sequences and quality scores were delivered in FASTQ files.

Clinical outcomes were evaluated using PASI, DLQI, Montgomery-Åsberg Depression Rating Scale-Self-report (MADRS-S), and HADS scores, assessed at each sampling point [6,7,9,10].

### 4.3. Bioinformatics

Bioinformatics was conducted according to the protocol of Eurofins Genomics (Konstanz, Germany). Ambiguous reads and chimeric sequences were removed using the UCHIME de novo algorithm [36] in the VSEARCH package version 2.3.0 [37]. The remaining reads were partitioned into Operational Taxonomic Units (OTUs) using Minimum Entropy Decomposition [38,39]. Sequences with low abundances (<0.02%) were also removed. 

DC-MEGABLAST assigned taxonomic information to each OTU by aligning cluster representative sequences with reference sequences, requiring a minimum of 70% sequence identity across at least 80% of the representative sequence. Further processing and taxonomic assignments were performed using QIIME (version 1.9.1). Abundances of bacterial taxonomic units were normalized using lineage-specific copy numbers of the relevant marker genes to improve estimates [40]. The taxonomic assignment was completed down to the lowest possible rank.

### 4.4. Statistical Analysis

Beta diversity analysis was conducted to examine the differences in OTU compositions between treatment groups and individual patients’ gut microbiota. Bray–Curtis dissimilarity values were generated from the OTU compositions, with a minimal relative abundance of 0.5% for inclusion in the analysis. To determine the statistical significance of differences between treatment groups and individual patients’ gut microbiota, PERMANOVA (a multivariate extension of ANOVA) was performed on the Bray–Curtis dissimilarity values. We performed pairwise PERMANOVA to assess the significance of gut microbiota differences before and after transitioning to brodalumab, while accounting for within-patient variations. Alpha diversity analysis (Shannon–Wiener diversity index) was conducted using OTU counts as input data, incorporating both species richness (number of distinct OTUs) and species evenness (relative abundance of each OTU). Pairwise comparisons using Wilcoxon signed rank test were performed on the alpha diversity values to determine the significance of differences between treatment groups by considering the variations within individual patients. All statistical analyses, including alpha diversity and beta diversity calculations, were performed using the vegan version 2.6.4 and pairwiseAdonis package in R version 0.4.1. 

Principal Coordinate Analysis (PCoA) was conducted by first transforming OTU counts to log-ratios, followed by the computation of distances between samples using the Euclidean distance metric. The ape package in R version 5.7.1 was employed to obtain the PCoA coordinates, which represent the samples in a reduced-dimensional space while preserving the dissimilarity relationships between them. The resulting coordinates were visualized in a two-dimensional PCoA plot, revealing the patterns of similarity and dissimilarity between OTUs in the gut microbiota of patients before and after switching to brodalumab. Differential Abundance Analysis (DAA) was performed using the R/Bioconductor package DESeq2 version 1.38.3 to identify significant changes in specific taxa at the phylum, genus, and species levels. All *p*-values were corrected for false discovery rates (FDRs) using the Benjamini–Hochberg method, with FDR < 0.1 as the significance threshold. Spearman correlation was applied to correlate the relative abundance of taxa with clinical variables (PASI, DLQI, HADS, and MADRS-S) using the Benjamini–Yekutieli method to adjust for multiple testing. R version 3.5.3 (The R Foundation for Statistical Computing, Vienna, Austria) was used for the correlation analysis, and adjusted *p* < 0.05 was considered significant.

## 5. Conclusions

This study is one of the first pilot investigations into the impact of switching biological agents from TNF-α inhibitors to brodalumab on the gut microbiota. Across all groups, Bacillota (Firmicutes) and Bacteroidota (Bacteroidetes) were the most prominent phyla. The gut microbiota did not experience any significant change after the switch, and no clinical variables correlated to specific phyla. While the Bacillota/Bacteroidota (Firmicutes/Bacteroidetes) ratio was stable overall, some patients experienced major shifts, suggesting that short-term changes may be limited and that the skin lesions heal faster than the gut microbiota alters. Dysbiosis and the worsening of symptoms seem to be driven more by the interplay and various ratios of different microbial populations than specific taxa. Given that the gut microbiota does not change significantly in the short term, brodalumab could potentially be used more freely as a rescue treatment in the future. However, it is important to notice that while the changes were not significant, some changes still occurred, and it is not possible to conclude if the gut microbiota became more dysbiotic or not due to the lack of a control group, calling for further studies to assess if brodalumab can be used as a rescue treatment or not. The gut microbiota has the potential to become a central component in deciding treatment for psoriasis patients in the future. Thus, as one of the pioneering studies on the impact of switching biological agents on the gut microbiota, this study lays the foundation for more comprehensive and in-depth research to follow.

## Figures and Tables

**Table 1:** Patient characteristics and demographics.

Variable	Sub Variable	Value
Age (years)	Mean (SD ^1^)	49.6 (11.3)
	Range (median)	30–70 (50.5)
Sex n ^2^ (%)	Female	6 (30)
	Male	14 (70)
Ethnicity n ^2^ (%)	Hispanic or Latino	1 (5.0)
	Not Hispanic or Latino	19 (95)
Mean BMI (SD ^1^)		30 (4.3)
Smokers (n ^2^)		3
First psoriasis diagnosis (age)	Mean (SD ^1^)	21.8 (10.1)
	Range (median)	11–44 (17.5)
Scalp involvement [n ^2^ (%)]	Yes	13 (65)
	No	7 (35)
Nail involvement [n ^2^ (%)]	Yes	8 (40)
	No	12 (60)
Psoriatic arthritis diagnosis n (%)	Yes No	3 (15) 17 (85)
Depression diagnosis n ^2^ (%)		2 (10)
PASI	Mean (SD ^1^)	9.3 (3.5)
DLQI	Mean (SD ^1^)	10.3 (7.2)
HADS	Mean (SD ^1^)	4.3 (2.9)
MADRS	Mean (SD ^1^)	7.3 (6.0)

^1^ Standard deviation. ^2^ Number.

**Table 2:** Spearman correlation of phyla and clinical variables presented as unadjusted and adjusted for multiple testing.

Phyla	PASI (Adjusted)	DLQI (Adjusted)	MADRS-S (Adjusted)	HADS (Adjusted)
F/B-ratio ^1^	0.08 (1.00)	0.66 (1.00)	0.30 (1.00)	0.69 (1.00)
Bacillota (Firmicutes)	0.10 (1.00)	0.98 (1.00)	0.38 (1.00)	0.92 (1.00)
Bacteroidota (Bacteroidetes)	0.30 (1.00)	0.83(1.00)	0.50 (1.00)	0.84 (1.00)
Actinomycetota (Actinobacteria)	0.59 (1.00)	0.93 (1.00)	0.33 (1.00)	0.78 (1.00)
Pseudomonadota (Proteobacteria)	0.34 (1.00)	0.15 (1.00)	0.42 (1.00)	0.56 (1.00)
Verrucomicrobiota (Verrucomicrobia)	0.97 (1.00)	0.45 (1.00)	0.58 (1.00)	0.39 (1.00)

^1^ Bacillota/Bacteroidota (Firmicutes/Bacteroidetes) ratio.

## Data Availability

Data available on request due to restrictions. Data from the study on group level are publicly available. However, individual patient data are protected due to ethical reasons.
